# Cognitive Reappraisal in Children: Neuropsychological Evidence of Up-Regulating Positive Emotion From an ERP Study

**DOI:** 10.3389/fpsyg.2019.00147

**Published:** 2019-02-11

**Authors:** Wen Liu, Fang Liu, Liang Chen, Zhongqing Jiang, Junchen Shang

**Affiliations:** ^1^College of Psychology, Liaoning Normal University, Dalian, China; ^2^School of Marxism, University of Science and Technology Liaoning, Anshan, China

**Keywords:** emotion regulation, positive emotion, LPP, children, cognitive reappraisal

## Abstract

Emotion regulation is a critical mechanism in the socio-emotional development of children. Previous studies revealed that children use cognitive reappraisal to downregulate negative emotions. Moreover, the amplitude of late positive potential (LPP) shows a more obvious reduction following neutral interpretations than following negative interpretations. However, whether children can use cognitive reappraisal to regulate positive emotions remains unclear. In the present study, 46 8- to 12-year-old children were asked to reappraise the meaning of pleasant pictures. Electroencephalography (EEG) data were collected during the task. As predicted, LPP amplitudes increased more following reappraisal condition than following pleasant condition. The analysis of spatial-temporal shifting patterns showed that the effect occurred in the earlier window for the posterior region. As time progressed, this effect evidenced a trend from posterior region to the central and anterior regions, especially for the younger children. Furthermore, the greater brain activations occurred in left hemisphere when children upregulated positive emotions which partially supported previous research suggesting that increasing positive emotion engaged primarily left-lateralized prefrontal regions. Taken together, the findings suggest that children can use cognitive reappraisal to upregulate positive emotions.

## Introduction

Emotion regulation refers to the internal and external processes that individuals conduct to monitor, evaluate, and revise emotional responses (Gross, 1998a). Emotion regulation plays an important role in the socio-emotional development of children (Cole et al., 2004). Considerable evidence in developmental studies indicate that the ability to regulate emotions increases with age (DeCicco et al., 2012; Zimmermann and Iwanski, 2014). Furthermore, emotion regulation is closely linked to academic performance, and successful emotion regulation is a potential predictor of positive emotional development in the future (Gross and John, 2003; Tugade and Fredrickson, 2007; Mega et al., 2014). Emotion regulation can be manifested in various forms, and most emotion regulation efforts aim to decrease negative emotions and enhance positive emotions for good mental health (Aldao et al., 2010; Gross, 2013).

Emotion regulation involves complex cognitive processing. In daily life, cognitive emotion regulation occurs when emotional information interacts with cognitive control. Cognitive emotion regulation refers to cognitive responses to emotional events, including consciously or unconsciously attempting to change individual emotional experiences, events, and (or) emotional types (Garnefski et al., 2001; Aldao et al., 2010; Livingstone and Srivastava, 2012; Liu et al., 2015).

Cognitive reappraisal is an effective emotion regulation strategy in adults studies (Mauss et al., 2007; Buhle et al., 2014). Cognitive reappraisal refers to the new meaning given by an individual to emotional events that consequently changes the understanding of emotional events (McRae et al., 2012a). Reappraisal strategies are frequently associated with positive outcomes, such as reducing anxiety and promoting well-being (Martin and Dahlen, 2005; McRae et al., 2012b). Despite significant research interested in cognitive reappraisal strategy among adults, few studies discussed this strategy in children. One reason is that children are still in the development stage, and their cognitive abilities are still developing; thus, they find difficulties in adapting cognitive strategies to regulate their emotions effectively (DeCicco et al., 2012). As they develop, children are inclined to adopt a cognitive emotion regulation strategy. Parent-reported questionnaire studies found that preschoolers could use cognitive reappraisal to adjust their negative emotional responses (Cole et al., 2009). In another cross-sectional study, the results of the use of self-reported questionnaires suggested that anxious children could reduce their negative emotions following cognitive reappraisal and that children with anxiety disorders were less likely to adopt cognitive reappraisal strategies (Carthy et al., 2010). Although cognitive reappraisal reduces negative emotions effectively, whether age-related differences exist in childhood and whether physiological arousal is effectively changed by this strategy require further investigation.

Given their high temporal resolution characteristics, event-related potentials (ERPs) are frequently used to investigate the processing of emotional events. Among adults, several studies indicated that late positive potential (LPP) is associated with emotion regulation (Schupp et al., 2000; Moran et al., 2013). LPP is typically detected over centro-parietal sites, appearing at approximately 300 to 2000 ms after stimulation, and is considered as an indicator of increasing attention following emotional stimuli (Cuthbert et al., 2000). The amplitude of the LPP reflects the degree of attention to individual emotional events, such that emotional stimuli (e.g., videos, faces, and pictures) elicit larger LPP than neutral stimuli (Cuthbert et al., 2000; Foti and Hajcak, 2008). LPP is also related to individual initiative to increase or decrease emotional responses. Specifically, LPP amplitudes decrease when individuals make a reappraisal on negative events (Hajcak and Nieuwenhuis, 2006) and increase when individuals enhance their positive emotions (Krompinger et al., 2008). In addition, the LPP was maximal at posterior recording sites in the early window, and shifted to central and anterior sites during the middle and late windows, moreover, in adult research, Kim and Hamann (2007) suggested that increasing emotions engaged primarily left hemisphere especially the prefrontal regions, whereas decreasing emotions engaged bilateral regions.

Although great interest lies in adult research, studies on children have emerged. Dennis and Hajcak (2009) found that LPP could serve as an electrophysiological marker for emotion regulation in children. School-age children (5–10 years old) could effectively use cognitive reappraisal to manage their negative emotions well. The LPP is notably small following reduced negative interpretations at posterior recording sites. However, the age differences in the processes of emotion regulation during development remain unclear. In response to the above research, DeCicco et al. (2012) investigated whether LPP is sensitive to cognitive reappraisal among 5- to 7-year-old children. They found no significant effect of reappraisal on LPP amplitudes, although they confirmed that LPP is sensitive to children with fear and anxiety. The research emphasized that such finding may be due to the children developing their cognition. Emotion regulation is therefore not utilized effectively in cognitive emotion regulation strategies.

Given that LPP could be used as an electrophysiological signature of cognitive emotion regulation in children (Lewis et al., 2006; Kujawa et al., 2013), whether LPP is an effective neurophysiological marker for upregulation in children necessitates critical evaluating. Neuroimaging studies have documented the differences in neural bases between positive and negative emotions. An fMRI study involving adults indicated that prefrontal regions and the left insula are significantly activated when people regulate positive emotions, whereas the activities of the left orbitofrontal gyrus and anterior cingulate cortex (ACC) are associated with regulating negative emotions (Mak et al., 2009). According to the bivariate model, positive and negative emotions are two independent variables, and the process of emotion regulation is different (Cacioppo and Berntson, 1994). People can upregulate or downregulate emotions according to personal goals (Larsen et al., 2001). For example, anxious people would inhibit the arousal of positive stimuli, whereas people who pay attention to positive emotional information in daily life, which would also facilitate downregulation of negative emotions (Jazaieri et al., 2015). Moreover, increasingly experiencing positive emotions promotes individual well-being and mental health (Hu et al., 2014). Fredrickson (2004) broaden-and-build model of positive emotions underline the extended effects of positive emotions on individual thinking and action. Reappraisal could promote individual positive emotional experience, and enhance the ability to regulate emotions. Neuroscience revealed that different emotional processing ways in dopaminergic neurons result in individual differences in the neuro loop function, which increases the tendency of children to become active (Lamm and Lewis, 2010).

Thus, the present study primarily aims to create a design that would examine whether children could use cognitive reappraisal to upregulate positive emotions, with LPP serving as an effective neural marker. In the present study, children conferred the pictures with another meaning or just viewed them. When the children required reappraisal, voice guidance was used to induce children to make cognitive reappraisal. On the basis of previous studies (DeCicco et al., 2012; Hua et al., 2015), we divided three time windows (300–600, 600–1000, and 1000–1500 ms) and divided the three regions (posterior, central, and anterior) and two hemispheres (left vs. right) of the brain to investigate the patterns of LPP amplitudes in different regions and confirm the occurrence of positive emotion regulation. We explored whether age differences exist in LPP amplitudes. Previous studies indicated that children from preschool age to adolescence could use cognitive reappraisal to regulate negative emotions effectively, but few studies examined the relationship between LPP and positive emotion regulation (Dennis and Hajcak, 2009; DeCicco et al., 2012). Moreover, individual differences in age require further discussions, we selected 8- to 12-year-old children in this study to examine whether children could apply cognitive reappraisal to upregulate positive emotions.

According to previous studies (Dennis and Hajcak, 2009; Hua et al., 2015), we tested two hypotheses in a group of 8- to 12-year-old children. (a) Children could use the cognitive reappraisal strategy to upregulate positive emotion effectively. The LPP amplitudes for pleasant pictures were smaller than those following cognitive reappraisal condition. (b) In addition, we predicted that reappraisal effect (LPP amplitudes for the cognitive reappraisal condition vs. pleasant picture condition) was positively associated with age.

## Materials and Methods

### Participants

Forty-six children participated in the ERP experiment. Of the 50 children, two children were excluded because they were unable to understand instructions. Another two children were excluded due to excessive movement artifacts. The final sample included 46 children (24 boys) between the ages of eight and twelve (*M* ±*SD* = 119.72 ± 15.57, range 96–146, in months). The breakdown is as follows: 12 children were 8 years old, 8 children were 9 years old, 11 children were 10 years old, 11 children were 11 years old, and 4 children were 12 years old. All children were recruited in a primary school in Dalian China. Their parents submitted an informed written consent prior to the experiment. All children received a gift after the experiment. Ethical approval was obtained from the Ethics Committee of Liaoning Normal University.

### Stimuli

Stimuli were 22 pleasant and 22 neutral pictures from the International Affective Picture System (IAPS; Lang et al., 2005)^1^. All pictures were evaluated by 20 other children (11 boys and 9 girls, aged 8–12 years) for valence and arousal using a five-point scale ranging from 1 (very unpleasant) to 5 (very pleasant) and 1 (very calm) to 5 (very exciting), respectively. The pleasant pictures had a mean valence of 4.64 (*SD* = 0.48) and an arousal of 4.21 (*SD* = 0.66). The neutral pictures had a mean valence of 3.70 (*SD* = 0.20) and an arousal of 1.80 (*SD* = 0.19). The ratings of pleasant and neutral pictures showed differences in valence [*t*_(19)_ = 8.48, *p* < 0.001], and arousal [*t*_(19)_ = 16.46, *p* < 0.001]. Two pleasant and two neutral pictures were used for practice. All the pictures displayed were colored and occupied a 14-inch computer monitor. Children were seated 60 cm from the computer during the task.

### Procedures

**FIGURE 1:**
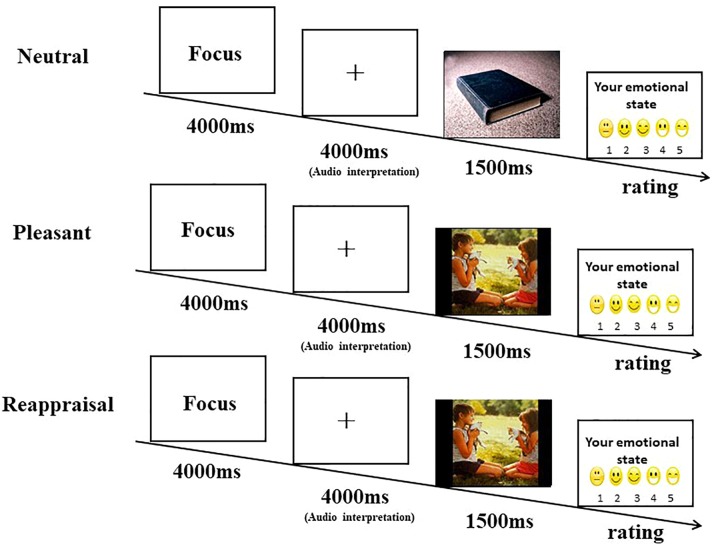
Schematic representation of the experimental procedure.

Children were seated in a dimly lit room approximately 60 cm in front of a computer monitor. Stimuli were presented using E-prime 1.0 software (Psychology Software Tools, Inc., Pittsburgh, PA, United States) on a black screen (1024 × 768 pixels). Prior to the ERP experiment, children had a behavior task similar to the ERP task. According to a previous study (DeCicco et al., 2014), children were told that they were participating in a game, and that they would hear a story for a few seconds prior to seeing an emotional picture presented. The children were also asked to match the listening story with the picture. They were also told to stay still and look at the screen. In the present study, we employed a modified paradigm. Children were given the instructions: “Listen to the words and think of the pictures so that they match the words. Try to match the words to the picture.” When the children made sense of the instructions, the task was initiated. Every trial started with a black screen that displayed the phrase, “Please pay attention, the game will begin soon.” The display lasted 4000 ms to capture the attention of the children. Then, the phrase was replaced by a fixation cross for 4000 ms, children were made to listen to an audio recording of either a neutral or pleasant interpretation of the picture. Specifically, for the neutral picture, the children heard a neutral description, such as “a book” (neutral picture following neutral interpretation, neutral condition). For the pleasant picture, the children were presented a neutral description, such as “a clown” (pleasant picture following neutral interpretation, pleasant condition), or a positive description, such as “the clown is performing for you” (pleasant picture following positive interpretation, reappraisal condition). A young male broadcaster recorded all the vocal interpretations in a recording studio. To ensure that the children heard all the vocal interpretations, we provided the children with earphones during the experiment. The volume was set to moderate. In the next screen, after the picture was presented for 1500 ms, the children were asked to rate the picture on a five-point scale ranging from 1 (very calm) to 5 (very exciting). In the next screen, the picture was presented for 1500 ms. After the picture was presented, the children were asked to rate the picture on a five-point scale ranging from 1 (very calm) to 5 (very exciting). Figure 1 shows the experimental procedure. Six practice trials (two pictures for each condition) were conducted before the start of the formal task to ensure that the children understood the task.

Each picture was displayed twice. The order of picture presentation was random in each block. The three condition blocks (neutral condition, pleasant condition, and reappraisal condition) were counterbalanced.

### EEG Recording

EEG data were recorded using 64 channels (EGI, Eugene, OR, United States). Reference electrodes were placed on the vertex channel (Cz), and two electrodes were placed on the left and right mastoids. The electrooculogram (EOG) was recorded from two vertical facial electrodes (above and below the left eye) and two horizontal facial electrodes (on the right side of the right eye and on the left side of the left eye). The EEG was amplified using a 0.1–100 Hz band-pass and sampled at 250 Hz/channel. All electrode impedances were maintained below 50 kΩ (Ferree et al., 2001). All offline data were computed using NetStation (EGI, Eugene, OR, United States). Eye movement artifacts (blinks and eye movements) were corrected offline and a 30-Hz low-pass filter was used. In artifact detection, the EEG data were contaminated by ocular artifacts (e.g., eye blinks and eye movements), and the mean EOG voltage exceeding ±50 μV was excluded from further analysis. The analysis window was from 200 ms before stimulus presentation to 1500 ms after stimulus presentation. According to previous studies, mean LPP amplitudes were computed within each of the following time windows on the basis of a visual inspection of the data: early (300–600 ms), middle (600–1000 ms), and late (1000–1500 ms) (Dennis and Hajcak, 2009; DeCicco et al., 2012). There were two regional clusters: left vs. right hemisphere, posterior vs. central vs. anterior. The left/right posterior clusters included electrodes P1/P2, P3/P4, PO3/PO4; the left/right central clusters included FC1/FC2, C1/C2, CP1/CP2; and the left/right anterior clusters included F1/F2, F3/F4, AF3/AF4.

### Data Analysis

We followed three steps for analysis. First, to examine the effect of positive cognitive appraisal in the behavior study, we used one-way ANOVA with repeated measures. For ERPs, analyses were conducted for each time window, and given that the sample size and age range, we split the sample into two groups (age 120 months and over, 24 children vs. under 120 months, 22 children). A 2 Age (younger children and older children) × 2 Hemisphere (left and right hemisphere) × 3 Region (posterior, central, anterior) × 3 Condition (view neutral pictures, view pleasant pictures, reappraisal for pleasant pictures) ANOVA with repeated measures was conducted to examine the upregulating effect. Emotion ratings were used as the dependent variables. Finally, we tested the associations between children’s age and the LPP reappraisal effect (reappraisal condition–pleasant condition).

The degrees of freedom and *p*-value were corrected according to the Greenhouse-Geisser method. Paired *t*-tests and Person correlations were conducted in the analyses. Partial eta squared represented the effect sizes in this study.

## Results

### Behavioral Results

The main effect was found for the three conditions (*F*_(2,90)_ = 144.70, *p* < 0.001, $\eta_{p}^{2}$ = 0.76), such that the ratings for the reappraisal condition were higher than the pleasant condition [*t*_(45)_ = 7.78, *p* < 0.001] and neutral condition [*t*_(45)_ = 14.03, *p* < 0.001]. Additionally, ratings were higher for the pleasant condition vs. neutral condition [*t*_(45)_ = 11.31, *p* < 0.001].

### ERP Results

To examine the time course of the LPP throughout the experiment and the brain regions where LPP occurred during emotion regulation, we identified three time windows, three regions and two hemispheres. The mean and standard deviations of the LPP amplitudes following three interpretations in each time window and region are presented in Table 1.

**Table 1:** Means and SD of LPP amplitudes following three conditions in three time windows and regions.

*Conditions*			
	*Neutral*	*Pleasant*	*Reappraisal*
*Time window*	*M(SD)*	*M(SD)*	*M(SD)*
Early (300–600 ms)			
Left/Right posterior	6.06/5.59 (3.30/3.04)	6.62/6.11 (3.00/2.77)	6.78/6.26 (2.91/2.68)
Left/Right central	−2.70/−2.49 (1.99/1.84)	−2.71/−2.50 (1.96/1.81)	−2.57/−2.37 (1.72/1.59)
Left/Right anterior	−5.41/−4.99 (2.85/2.63)	−5.00/−4.62 (2.68/2.47)	−4.69/−4.33 (2.58/2.38)
*Middle (600–1000 ms)*			
Left/Right posterior	2.16/2.00 (2.20/2.05)	2.26/2.09 (2.15/1.99)	2.29/2.21 (1.97/1.82)
Left/Right central	−0.56/−0.52 (1.72/1.59)	−0.04/−0.04 (1.59/1.47)	0.06/0.05 (1.27/1.17)
Left/Right anterior	−3.01/−2.78 (2.92/2.70)	−2.12/−1.96 (2.79/2.57)	−1.48/−1.37 (2.29/2.12)
*Late (1000–1500 ms)*			
Left/Right posterior	1.76/1.63 (1.58/1.46)	1.90/1.76 (1.40/1.30)	2.09/1.94 (1.40/1.30)
Left/Right central	−0.61/−0.56 (1.21/1.12)	−0.47/−0.43 (1.11/1.02)	−0.27/−0.25 (0.96/0.88)
Left/Right anterior	−1.98/−1.83 (1.84/1.70)	−1.71/−1.57 (1.85/1.71)	−1.41/−1.30 (1.62/1.50)

#### Early Window (300 to 600 ms)

A main effect of Hemisphere existed, [*F*_(1,44)_ = 9.09, *p* < 0.01, $\eta_{p}^{2}$ = 0.17], with the brain activations being higher for left hemisphere than for right hemisphere. A main effect of Condition existed, [*F*_(2,88)_ = 9.76, *p* < 0.001, $\eta_{p}^{2}$ = 0.18], with the amplitudes being larger for positive cognitive reappraisal than for pleasant pictures [*t*_(45)_ = 2.02, *p* < 0.05] and neutral [*t*_(45)_ = 3.87, *p* < 0.01]. Amplitudes for pleasant pictures were larger than those for neutral [*t*_(45)_ = 2.82, *p* < 0.001]. The analysis also showed a main effect of Region, [*F*_(1.43,88)_ = 225.37, *p* < 0.001, $\eta_{p}^{2}$ = 0.84]. The amplitudes were larger in the posterior recording sites than in the central [*t*_(45)_ = 16.22, *p* < 0.001] and anterior [*t*_(45)_ = 16.17, *p* < 0.001] recording sites. Moreover, the amplitudes for the central recording sites were larger than those for the anterior recording sites [*t*_(45)_ = 6.08, *p* < 0.001]. There was a significant interaction between Hemisphere and Region, [*F*_(2,88)_ = 225.37, *p* < 0.001, $\eta_{p}^{2}$ = 0.84]. Specifically, with the amplitudes being larger for the left-posterior recording sites than for right-posterior [*t*_(45)_ = 12.92, *p* < 0.001],and the amplitudes being larger for the left-central recording sites than for right-central [*t*_(45)_ = 13.10, *p* < 0.001], and the amplitudes being larger for the left-anterior recording sites than for right-anterior [*t*_(45)_ = 13.10, *p* < 0.001]. There was an also significant interaction between Hemisphere and Condition, [*F*_(2,79.1)_ = 9.76, *p* < 0.001, $\eta_{p}^{2}$ = 0.18]. Specifically, with the left hemisphere activations being higher for the positive cognitive reappraisal than neutral [*t*_(45)_ = 16.41, *p* < 0.001], and with the right hemisphere activations being higher for the positive cognitive reappraisal than neutral [*t*_(45)_ = 16.58, *p* < 0.001]. Age main effect and other interactions effects did not reach significance.

#### Middle Window (600 to 1000 ms)

Late positive potential was sensitive to condition, [*F*_(2,88)_ = 20.40, *p* < 0.001, $\eta_{p}^{2}$ = 0.32]. LPP following positive cognitive reappraisal was significantly larger than that following pleasant pictures [*t*_(45)_ = 2.77, *p* < 0.01] and neutral appraisal [*t*_(45)_ = 5.76, *p* < 0.001]. Amplitudes for pleasant pictures were larger than those for neutral appraisal [*t*_(45)_ = 3.72, *p* < 0.001]. A main effect was found in the region, [*F*_(1.29,88)_ = 62.81, *p* < 0.001, $\eta_{p}^{2}$ = 0.59], and the LPP amplitudes for the posterior recording sites were larger compared with those for the central [*t*_(45)_ = 7.13, *p* < 0.001] and anterior [*t*_(45)_ = 7.80, *p* < 0.001] recording sites. The amplitudes for the central recording sites were larger than those for the anterior recording sites [*t*_(45)_ = 5.85, *p* < 0.001]. A significant interaction existed between region and condition [*F*_(2.77,121.94)_ = 3.00, *p* < 0.05, $\eta_{p}^{2}$ = 0.06]. *Post hoc* comparisons at central sites confirmed that LPP was larger for the reappraisal condition than for the pleasant condition [*t*_(45)_ = 13.32, *p* < 0.001]. However, contrary to predictions, no differences occurred between the reappraisal and pleasant condition in the anterior region. There was a significant interaction between Hemisphere and Condition, [*F*(_2,88)_ = 20.40, *p* < 0.001, $\eta_{p}^{2}$ = 0.31]. Specifically, for the positive cognitive reappraisal, the left hemisphere activations were higher than the right hemisphere [*t*_(45)_ = 1.98, *p* < 0.05]. We also found the interaction effect between Age and Region, [*F*_(2,88)_ = 7.74, *p* < 0.001, $\eta_{p}^{2}$ = 0.15]. *Post hoc* comparisons at anterior sites confirmed that LPP was larger for the older children than for the younger children, [*t*_(45)_ = 7.23, *p* < 0.01]. Age main effect and other interactions effects did not reach significance.

#### Late Window (1000 to 1500 ms)

**FIGURE 2:**
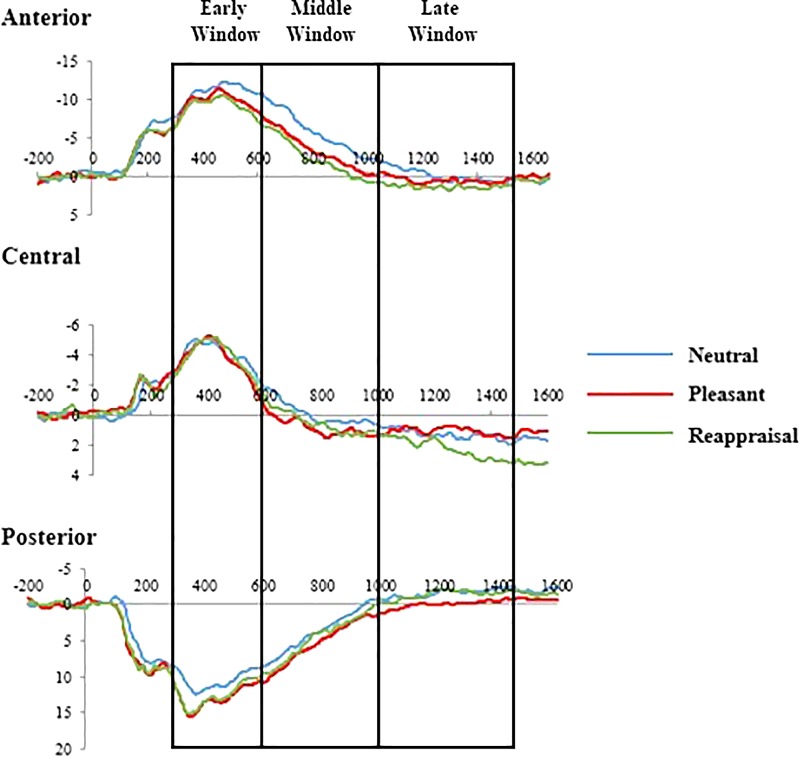
LPP in each region for neutral, pleasant pictures, and positive cognitive reappraisal condition.

A main effect of condition existed, [*F*_(2,88)_ = 10.23, *p* < 0.001, $\eta_{p}^{2}$ = 0.19]. The amplitudes for positive cognitive reappraisal were larger than those for pleasant [*t*_(45)_ = 2.02, *p* < 0.05] and neutral pictures [*t*_(45)_ = 3.02, *p* < 0.01]. The amplitudes for pleasant pictures were larger than those for neutral pictures [*t*_(45)_ = 4.05, *p* < 0.001]. The analysis showed a main effect of region, [*F*_(1.42,88)_ = 89.64, *p* < 0.001, $\eta_{p}^{2}$ = 0.67]. The amplitudes were larger in the central recording sites than in the posterior [*t_(_*_45)_ = 9.97, *p* < 0.001] and anterior recording sites [*t*_(45)_ = 9.63, *p* < 0.001], and the amplitudes for the posterior recording sites were larger than those for the anterior recording sites [*t*_(45)_ = 5.26, *p* < 0.001]. There was a significant interaction between Hemisphere and Region, [*F*_(2,88)_ = 89.64, *p* < 0.001, $\eta_{p}^{2}$ = 0.67]. Specifically, with the amplitudes being larger for the left-central recording sites than for right-central [*t*_(45)_ = 4.81, *p* < 0.001],and the amplitudes being larger for the left-anterior recording sites than for right-anterior [*t*_(45)_ = 13.10, *p* < 0.001]. There was a significant interaction between Hemisphere and Condition, [*F*_(2,88)_ = 10.23, *p* < 0.001, $\eta_{p}^{2}$ = 0.19], but simple effect evidenced no significance. We also found the interaction effect between Age and Region, [*F*_(2,88)_ = 5.69, *p* < 0.01, $\eta_{p}^{2}$ = 0.12]. *Post hoc* comparisons at anterior sites confirmed that LPP was larger for the older children than for the younger children, [*t*_(45)_ = 6.24, *p* < 0.01]. Age main effect and other interactions effects did not reach significance. Figure 2 shows the stimulus-locked ERPs at the posterior, central, anterior electrodes (the mean amplitude of the electrodes included in each region).

### The LPP and Age in Months

**FIGURE 3:**
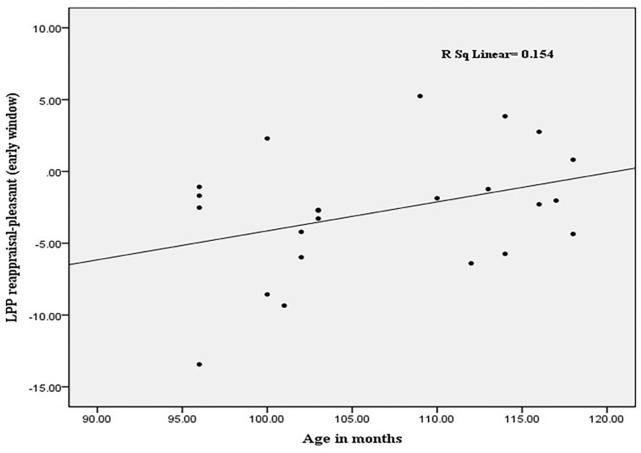
Increasing age in months was associated with a greater increase in mean LPP amplitudes in the reappraisal vs. pleasant condition (22 children).

In order to test whether the LPP amplitude of reappraisal increased with age, we examined the associations between age in months and LPP reappraisal effect of two groups, respectively. The results showed that increasing age in months was associated with large amplitudes due to reappraisal only in the under 120 months group [*r_(22)_* = 0.39, *p* < 0.05], and this correlation was significant for the early window. Figure 3 presents the correlations between age and reappraisal.

## Discussion

The present study demonstrated that children who used cognitive reappraisal could upregulate positive emotion. From the behavioral results, we found the main effect of conditions in which the ratings following the reappraisal condition were higher than those following the other conditions. This study indicated that cognitive reappraisal modulated LPP in the child participants. Moreover, the amplitudes of LPP following upregulating interpretations were larger those following pleasant and neutral pictures. In addition, pleasant pictures compared with neutral pictures evoked larger LPP amplitudes in the entire time course. Taken together, the findings suggest that 8- to 12-year-old children could use the cognitive emotion regulation strategy to upregulate positive emotions.

Early LPP is regarded as a typical ERP component in emotion regulation research; it is widely distributed over the posterior, central, and anterior scalp sites and reflects motivated attention (Olofsson et al., 2008). In the current study, the amplitudes for the posterior region were the largest among all the amplitudes for the three regions, especially for the left hemisphere. These results are consistent with previous studies involving children and preschoolers (DeCicco et al., 2012, 2014; Hua et al., 2015), in which the LPP amplitudes were maximal in the early window for the posterior region. We also found the main effect of Condition, highly motivated stimuli evoked larger LPP amplitudes compared with neutral stimuli (Righi et al., 2014), and therefore, the results may reflect the process of voluntary attention increasing emotional stimuli (Wentura et al., 2000). This process is a necessary condition for children to invest and participate in cognitive reappraisal (Hajcak et al., 2010). In addition, the results are partially consistent with previous studies on adults, in which LPP amplitudes for appraisal were in the maximal posterior regions in the 400–1000 ms time window (Foti and Hajcak, 2008). In the middle time window, LPP was larger for the reappraisal condition than for the pleasant condition at the central region, especially for the left hemisphere. The results agree with those of previous adult studies and suggest that the different descriptions of pictures caused LPP changes during reappraisal (Hajcak and Nieuwenhuis, 2006; Foti and Hajcak, 2008). Source location studies support the results; that is, LPP was detected over the centroparietal electrode sites (Cuthbert et al., 2000; Olofsson et al., 2008). Changes in LPP amplitudes were found in the late window; the amplitudes for the reappraisal condition were the largest among all the amplitudes for the three conditions, and the amplitudes were largest in the central recording sites among all the amplitudes for the three regions. We also found the interaction effect between Hemisphere and Region, the amplitudes being larger for the left-central and left-anterior recording sites than right brain region. The results partially supported the previous study which indicated that increasing positive emotion engaged primarily left-lateralized regions (Kim and Hamann, 2007). LPP in the late window reflected the process of using cognitive resources to regulate emotions (Dennis and Hajcak, 2009; Weinberg et al., 2013) and may indicate that children could modulate LPP via cognitive reappraisal. The results are similar to those of previous downregulating studies. Hua et al. (2015) found that LPP amplitudes following cognitive reappraisal are significantly reduced compared with those following negative interpretation condition among 4- to 6-year-old children.

Interestingly, the present study yielded some different results. DeCicco et al. (2014) found no regulating effect of LPP amplitudes and reported that 5- to 9-year old children are unable to downregulate negative emotions by cognitive reappraisal. In addition, another study of positive emotion regulation demonstrated that adults could modulate LPP amplitudes for pleasant pictures via cognitive reappraisal (Krompinger et al., 2008). LPP amplitudes attenuated following suppressed condition other than only passed view, thus suggesting that people could use the cognitive emotion regulation strategy to downregulate positive emotions; however, no effect of upregulating instruction was observed (Krompinger et al., 2008). In the present study, we found the upregulating reappraisal effect in the posterior and central regions. A possible explanation is that the instructions employed in DeCicco’s study lasted 5–7 s, which is too long for children, we improved the task and reduced the duration of vocal interpretation to less than 4 s, thereby alleviating the burden of working memory. However, we could not find the same effect in the anterior region, one possible explanation for the findings is that the lack of prefrontal lobe development in our sample compare to young adults (Giedd et al., 1999). On the other hand, it may reflect the fact that the upregulation and downregulation of positive emotions may have different mechanisms (Giuliani et al., 2008).

Our results are similar to previous downregulating studies on adults (Foti and Hajcak, 2008), young children (DeCicco et al., 2014), and preschool children (Hua et al., 2015). The amplitude of LPP was the largest for the posterior region in the early window and then shifted to the central and anterior regions in the late window (Moser et al., 2009). The analysis of general spatial–temporal patterns contributed to the comprehensive exploration of the influence of emotion regulation strategies (Wiens et al., 2011). In adult studies, emotional processing usually occurs in 400–1000 ms for the posterior/superior recording sites and shifts to the frontal lobe in 1000–2000 ms (Olofsson et al., 2008). Meanwhile, the amplitude of LPP is reduced in the occipital lobe and shows an upward trend in the frontal lobe, thereby suggesting that adults transfer cognitive resources from visual processing to cognitive control during emotion regulation (Moran et al., 2013). Results on young children and preschool children support this pattern (DeCicco et al., 2014; Hua et al., 2015). This finding is partially consistent with the present study. In the early and middle time windows, the amplitude of the LPP for the posterior region was larger than that for the middle and anterior regions. In the late time window, the amplitude for the anterior and central region increased, the amplitude for the posterior region attenuated, especially for the left brain region. However, compared with previous studies, this study still showed differences. In previous studies, the amplitudes of LPP for the anterior region in the late window are maximal in comparison with those for the central and posterior regions (Dennis and Hajcak, 2009; Hua et al., 2015). However, we failed to find consistent results. This limitation may be due to the lack of maturity in the development of the prefrontal lobe in children; therefore, young children have difficulty employing cognitive control to regulate emotions, consequently leading to the moderating effects of the gap (Lamm and Lewis, 2010). However, the main effect of the condition of LPP amplitude was observed throughout all time windows in conjunction with behavior results, thus suggesting that 8- to 12-year-old children could use cognitive reappraisal to upregulate positive emotions. Another interpretation for the present results is that processing positive and negative emotions is different, and compared with that of positive emotions, the arousal of negative emotions is stronger (Giuliani et al., 2008).

We also found the interaction effect between Age and Region in the middle and late windows. Older children showed larger LPP amplitudes in the anterior region, suggesting that the prefrontal lobe of older children may developed better than younger children. Additionally, reappraisal effect was associated with age. With increasing age, the children showed larger differences between the reappraisal and pleasant conditions, suggesting that reappraisal could change their evaluation of pleasant pictures. A shift in cognitive reappraisal abilities from 8 to 10 years was associated with cognitive and neural development. During this age period, the brain function associated with cognitive and working memory develops rapidly and could improve children’s reappraisal ability (Bunge and Wright, 2007). The results are similar to previous study (DeCicco et al., 2014), children aged 5- to 10-year-olds displayed reduced LPP amplitudes when they downregulated unpleasant pictures. However, this effect did not emerge for the older children, it may due to the brain development in the preadolescence tend to maturity (Giedd et al., 1999). The LPP in childhood is marked by increased engagement of upregulating pleasant pictures, with a shift to a relative stability of processing pattern in preadolescence. Taken together, these results suggest that LPP is a useful neural signature for emotion regulation competencies for children.

The current study has some limitations that should be considered. First, the sample size of our study was comparatively small. Although this sample size is similar to that in previous children studies (Dennis and Hajcak, 2009; DeCicco et al., 2014; Hua et al., 2015) and other adult studies (Moser et al., 2006; Schönfelder et al., 2013), the small number of children in our work restricted our ability to depict the trajectory of children’s LPP development. In addition, the current study did not consider the possible influence of family factors (e.g., maternal depression and parenting style). Future research should employ a comprehensive design to examine systematically the regulatory effects of LPP modulated by environment factors (Loman and Gunnar, 2010). Longitudinal studies are needed to confirm these findings. Second, although we measured children’s ERPs during the upregulation of emotions, other physiological information (e.g., heart rate and neuroimaging) should be examined in future research to reveal the mechanism of emotion regulation in children. Third, in the current study, the instructions for reappraisal were specific to children; however, we are not certain about whether implicit instructions are effective for reappraisal among adults (Gross, 1998b; Sheppes et al., 2014). The findings should be replicated using different instructions. Fourth, although we found the brain activations were greater in left hemisphere when children upregulated positive emotions, however, we could not replicate previous results which it found upregulating effect engaged primarily left-lateralized prefrontal region (Kim and Hamann, 2007). We could use neuroimaging technology to further examine the lateralization effect in the future. Finally, we could investigate whether the ability to upregulate positive emotions moderated by cultural factors in the future. For example, some studies have shown that Europeans and Americans appear better at enhancing emotional responses than East Asians (Tsai, 2007; Varnum and Hampton, 2017). However, in the present study, we found that children could use reappraisal strategy to upregulate positive emotions, one possible explanation for the findings was that our sample are all children, they may be less influenced by cultural factors than adults, more cross-cultural studies of children emotion regulation should be the direction for future research.

## Conclusion

The present study showed that for children, pleasant pictures evoked larger LPP than neutral pictures and that LPP amplitudes following positive interpretations were more enhanced than those following pleasant pictures; the effect was broadly distributed in the brain regions. In addition, results may suggest that cognitive reappraisal ability rapidly shifts during childhood. In this work, the effect was seemingly processed at earlier stages and then showed a shift in the distribution from the posterior region to all regions. Taken together, our findings indicate that children could upregulate LPP amplitudes via cognitive reappraisal.

## Author Contributions

LC and FL drafted the manuscript and performed the experiments. WL, ZJ, and JS provided critical revisions. LC and WL designed the experiments. LC, FL, and WL analyzed the data.

## Conflict of Interest Statement

The authors declare that the research was conducted in the absence of any commercial or financial relationships that could be construed as a potential conflict of interest.

## Footnotes

1 Pleasant pictures: 1440, 1500 (for practice), 1600, 1610, 1811, 1812, 1999, 2057, 2091, 2092, 2341, 2345, 2550, 2650, 2655, 5000, 5450, 5470, 5621, 5760, 7281, 7282. Neutral pictures: 7000, 7002 (for practice), 7004, 7006, 7020, 7025, 7031, 7050, 7060, 7080, 7090, 7095, 7140, 7150, 7186, 7190, 7211, 7217, 7233, 7235, 7545, 7950.
